# SnCl_2_/TiCl_3_-Mediated Deoximation of Oximes in an Aqueous Solvent

**DOI:** 10.3390/molecules17032464

**Published:** 2012-03-01

**Authors:** Mei-Huey Lin, Han-Jun Liu, Cheng-Yu Chang, Wei-Cheng Lin, Tsung-Hsun Chuang

**Affiliations:** Department of Chemistry, National Changhua University of Education, Changhua 50007, Taiwan

**Keywords:** deoximation, aqueous solvent, ketoxime, aldoxime, tin chloride, titanium trichloride

## Abstract

A simple procedure for SnCl_2_/TiCl_3_-mediated deoximation of ketoximes in an aqueous solvent is reported. Under the conditions developed in this effort, various ketones and aldehydes are produced in good to excellent yields.

## 1. Introduction

Oximes are often used in organic synthesis as protected forms of carbonyl compounds [1] or as carbonyl derivatives for purification and characterization purposes [2]. Furthermore, oximes can be prepared from noncarbonyl compounds, such as nitroalkanes [3,4,5], or primary amines [6], thus deoximation provides an alternative approach for the syntheses of aldehydes and ketones. A plethora of examples of procedures for the regeneration of carbonyl compounds from oximes have been reported. So far a good number of deoximation methods based on hydrolytic [7], reductive [8,9,10,11,12], oxidative [13,14,15,16,17,18,19], and transoximation [20,21,22] reactions have been developed. Among them some require strong acidic conditions; some take long reaction times, and give low product yields, while some are performed at higher temperatures. For example, regeneration of carbonyl compounds from oximes via direct hydrolysis usually involves strong acidic conditions due to the relatively high hydrolytic stability of oximes, and thus leads to the damage of acid-sensitive groups and the formation of amides as byproducts by Beckmann rearrangement [23,24]. Besides, most of reductive and oxidative methods require reagents that are often hazardous or very toxic, expensive or not readily available. Therefore, a milder, high yielding, and inexpensive method is still in demand.

Tin and tin-containing Lewis acids have been extensively used in organic chemistry owing to their low cost, commercial availability and modestly low toxicity [25,26]. Deoximation of oximes by using SnCl_2_-SiO_2_ has been reported. However, harsh reaction conditions are applied such as reflux or microwave irradiation at high temperature [27,28]. Several examples of mild methods that use tin metal and stannic chloride to promote allylation reactions of protected carbonyls such as enol ethers and acetals in aqueous media have been described [29,30,31,32]. Apparently, the protected carbonyls are hydrolyzed to the corresponding aldehydes, which then undergo allylation in the presence of allyl anion equivalents. In this regard, we envisioned that deoximation of oximes by tin-mediated hydrolysis reactions under aqueous and mild conditions should be possible. Below, we describe the results of a study which demonstrate that oximes serve as starting materials for tin promoted hydrolysis reactions.

## 2. Results and Discussion

The effort began with an investigation of metal- or metal halide-mediated aqueous deoximation of acetophenone oxime (**1a**). The conditions employed and the results of the reactions are presented in Table 1 [33].

**Table 1:** Metal- or metal halide-mediated deoximation of ketoxime ^a^. 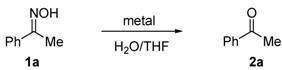

Entry	Metal	Time, conversion *^b^*
1	TiCl_3_	4 h, 12%
2	SnCl_2_	4 h, 14%
3	SnCl_2_/TiCl_3_	4 h, 99+%
4	SnCl_2_/KI	4 h, 14%
5	Sn/TiCl_3_	4 h, 80%
6	Mn/TiCl_3_	4 h, 23%
7	In/TiCl_3_	4 h, 55%
8	Fe/TiCl_3_	4 h, 53%
9	Cu/TiCl_3_	4 h, 39%
10	Zn/TiCl_3_	4 h, 56% *^c^*
11 *^d^*	TiCl_3_	4 h, 92%
12 *^d^*	SnCl_2_	4 h, 26%

^a^ Conditions: **1a** (1.0 mmol) and indicated metal or metal halide (1.5 mmol) in THF (1.0 mL)/water (1.0 mL) at r.t.; ^b^ conversion was determined by ^1^H-NMR; ^c^ Isolated yield; ^d^ Conditions: **1a** (1.0 mmol) and indicated metal halide (3.0 mmol) in THF (1.0 mL)/water (1.0 mL) at r.t.

The results show that SnCl_2_/TiCl_3_ serves as a superior reagent for the deoximation of acetophenone oxime (**1a**) in aqueous medium (Table 1, entry 3). It is reported that SnCl_2_ is prone to partial hydrolysis in water and generates an acidic solution [34]. However, the results showed that the acidic conditions generated are not sufficient to promote regeneration of carbonyl compounds from ketoxime in aqueous solvent (Table 1, entries 2, 4 and 12). The reduction of oximes to form imines, using trivalent titanium reductant or utilizing a low-valent titanium reagent generated from TiCl_4_/SnCl_2_ has been described [35,36,37,38]. However, these reactions are performed under anhydrous conditions and excess amounts of reagents are employed. Thus, we envisaged that imines formed in this manner would be susceptible to rapid hydrolysis to produce ketones. Herein, a commercially available titanium trichloride 20% in 3% hydrochloric acid was employed in this study. The intervention and effect of this reduction process are seen by comparing the rates of reactions promoted by using various low-valent titanium reagents (Table 1, entries 1, 3 and 5–11). It is important to note that the deoximation process was deteriorated by using low-valent titanium generated from zinc (Table 1, entry 10).

**Table 2:** SnCl_2_/TiCl_3_-mediated deoximation of ketoximes *^a^*. 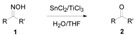

Entry	Substrate	Time, yield	Entry	Substrate	Time, yield
1	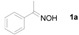	4 h, 96%	9	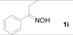	5 h, 89%
2	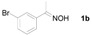	6 h, 92%	10	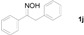	9 h, 97%
3	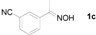	9 h, 92%	11	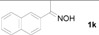	6 h, 97%
4	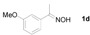	4 h, 84%	12	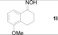	9 h, 99%
5	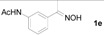	9 h, 82%	13	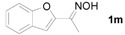	9 h, 83%
6	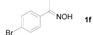	9 h, 98%	14	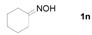	3 h, 91%
7	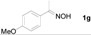	3 h, 95%	15	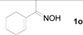	7 h, 92%
8	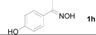	2.5 h, 94%			

^a^ Conditions: ketoxime **1** (1.0 mmol), SnCl_2_ (1.5 mmol) and TiCl_3_ (1.5 mmol) in THF (1.0 mL)/water (1.0 mL) at r.t.

Next, the substrate scope of the deoximation reactions of ketoximes was probed under the SnCl_2_/TiCl_3_-mediated conditions (Table 2). It is found that the amount of TiCl_3_ could be lowered but it depends on the substrate. For example, a catalytic amount of TiCl_3_ (0.25 equivalent) accompanied with SnCl_2_ (1.0 equivalent) was able to deoximate acetophenone oxime (**1a**) but at least one equivalent of TiCl_3_ was required to deoximate oxime **1k**. Therefore, we chose fixed amounts of SnCl_2_ and TiCl_3_ (1.5 equivalents each) as a general procedure for deoximation of various ketoximes shown in Table 2. As can be seen by viewing the results displayed in Table 2, the yields of these processes starting with both aromatic (Table 2, entries 1–13) and aliphatic (Table 2, entries 14 and 15) oximes, are good to excellent. Interestingly, the presence of an amide group that is present in **1e** (Table 2, entry 5) and a free phenol group that is present in **1h **(Table 2, entry 8) do not alter the efficiency of the reaction. In addition, aromatic halogen (**1b**, **1f**), nitrile (**1c**), and alkoxyl (**1d**, **1g**, **1l**) as well as olefin (**1o**) were tolerated under this mild condition.

With the excellent results from deoximation of ketoximes, we turned our attention to deoximation of aldoximes with the same approach and the results are shown in Table 3. Deoximation worked well for aldoximes **1p**, **1q**, and **1r** with the SnCl_2_/TiCl_3_ system (entries 3, 6 and 9, Table 3) and deoximation products were obtained in excellent yields under this mild condition. Due to poor solubility of aldoxime **1r** in water, THF is added to boost the reaction to complete (entry 9 *vs.* entry 10, Table 3).

**Table 3:** Metal halide-mediated deoximation of aldoxime *^a^*. 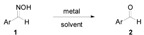

Entry	Substrate	Metal	Solvent	Time	Conversion *^b^* (%)
1	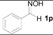	TiCl_3_	H_2_O	2 h	33
2	**1p**	SnCl_2_	H_2_O	2 h	4
3	**1p**	SnCl_2_/TiCl_3_	H_2_O	2 h	99 (96) *^c^*
4	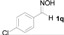	TiCl_3_	H_2_O	5 h	12
5	**1q**	SnCl_2_	H_2_O	5 h	<1
6	**1q**	SnCl_2_/TiCl_3_	H_2_O	5 h	99 (93) *^c^*
7	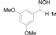	TiCl_3_	THF/H_2_O (1/3)	4 h	17
8	**1r**	SnCl_2_	THF/H_2_O (1/3)	4 h	9
9	**1r**	SnCl_2_/TiCl_3_	THF/H_2_O (1/3)	4 h	99 (97) *^c^*
10	**1r**	SnCl_2_/TiCl_3_	H_2_O	6 h	71

^a^ Conditions: aldoxime **1** (1.0 mmol) and indicated metal halide (1.0 mmol) in solvent (2.0 mL) at r.t.; ^b^ Conversion was determined by ^1^H-NMR; ^c^ Isolated yield of **2**.

## 3. Experimental

### 3.1. General Information and Materials

All commercially available chemicals were used without further purification. Oximes were prepared according to the reported procedures [39]. TLC analyses were run on a glass plate (Silica gel 60 F254) and were visualized using UV or a solution of phosphomolybdic acid in ethanol (5 wt%) or *p*-anisaldehyde stain. Flash chromatography was performed using silica gel (70–230 mesh). ^1^H and ^13^C-NMR spectra were recorded on a 300 MHz spectrometer (Bruker AV-300). Chemical shifts are reported relative to CHCl_3_ [δH 7.24, δC (central line) 77.0]. Mass spectra and high-resolution mass spectra were recorded under electron spray interface (ESI) conditions (Finnigan/Thermo Quest MAT).

### 3.2. General Procedure for SnCl_2_/TiCl_3_-Mediated Deoximation of Ketoximes in an Aqueous Solvent

Tin chloride dihydrate (345 mg, 1.5 mmol) and titanium(III) chloride, (0.96 mL, 1.5 mmol, 20% in 3% hydrochloric acid) were added successively to a solution of oxime **1** (1.0 mmol) in THF (1 mL)/water (1 mL) at r.t. The mixture was stirred at ambient temperature until all the starting material was consumed, the solution was extracted with Et_2_O (3 × 5 mL). The combined organic layers were dried over MgSO_4_, filtered, and concentrated *in vacuo*, giving a residue which was subjected to silica gel chromatography to furnish the pure ketone **2**.

*Acetophenone* (**2a**). Following the general procedure, the title compound was obtained (115 mg, 96%). Oil; TLC (EtOAc/hexanes (1:2)) *R f*= 0.58; ^1^H-NMR (CDCl_3_): δ 2.58 (s, 3H), 7.41–7.54 (m, 3H), 7.93 (dd, *J* = 8.4, 1.2 Hz, 2H). Data are in good agreement with the literature [40].

*1-(3-Bromophenyl)ethanone* (**2b**). Following the general procedure, the title compound was obtained (183 mg, 92%). Oil; TLC (EtOAc/hexanes (1:4)) *R f*= 0.68; ^1^H-NMR (CDCl_3_): δ 2.57 (s, 3H), 7.33 (t, *J* = 7.8 Hz, 1H), 7.65–7.69 (m, 1H), 7.84–7.87 (m, 1H), 8.06 (d, *J* = 2.1 Hz, 1H). Data are in good agreement with the literature [41].

*3-Acetylbenzonitrile* (**2c**). Following the general procedure, the title compound was obtained (134 mg, 92%). Oil; TLC (Et_2_O/hexanes (1:2)) *R f*= 0.25; ^1^H-NMR (CDCl_3_): δ 2.62 (s, 3H), 7.59 (t, *J* = 7.8 Hz, 1H), 7.80–7.84 (m, 1H), 8.14–8.21 (m, 2H). Data are in good agreement with the literature [42].

*1-(3-Methoxyphenyl)ethanone* (**2d**). Following the general procedure, the title compound was obtained (126 mg, 84%). Oil; TLC (EtOAc/hexanes (1:4)) *R f*= 0.75; ^1^H-NMR (CDCl_3_): δ 2.58 (s, 3H), 3.84 (s, 3H), 7.07–7.11 (m, 1H), 7.35 (t, *J* = 7.8 Hz, 1H), 7.46–7.53 (m, 2H). Data are in good agreement with the literature [43].

N*-(3-Acetylphenyl)acetamide* (**2e**). Following the general procedure, the title compound was obtained (145 mg, 82%). Oil; TLC (EtOAc/hexanes (1:2)) *R f*= 023; ^1^H-NMR (CDCl_3_): δ 2.17 (s, 3H), 2.52 (s, 3H), 7.33 (t, *J* = 7.8 Hz, 1H), 7.59 (d, *J* = 7.8 Hz, 1H), 7.91 (d, *J* = 7.8 Hz, 1H), 8.05 (s, 1H), 8.85 (s, 1H). Data are in good agreement with the literature [44].

*1-(4-Bromophenyl)ethanone* (**2f**). Following the general procedure, the title compound was obtained (195 mg, 98%). Oil; TLC (EtOAc/hexanes (1:4)) *R f*= 0.53; ^1^H-NMR (CDCl_3_): δ 2.56 (s, 3H), 7.57 (d, *J* = 7.8 Hz, 2H), 7.78 (d, *J* = 7.8 Hz, 2H). Data are in good agreement with the literature [45].

*1-(4-Methoxyphenyl)ethanone* (**2g**). Following the general procedure, the title compound was obtained (143 mg, 95%). Oil; TLC (EtOAc/hexanes (1:9)) *R f*= 0.43; ^1^H-NMR (CDCl_3_): δ 2.52 (s, 3H), 3.81 (s, 3H), 6.86 (d, *J* = 7.8 Hz, 2H), 7.87 (d, *J* = 7.8 Hz, 2H). Data are in good agreement with the literature [46].

*1-(4-Hydroxyphenyl)ethanone* (**2h**). Following the general procedure, the title compound was obtained (128 mg, 94%). Oil; TLC (Et_2_O/hexanes (1:2)) *R f*= 0.58; ^1^H-NMR (CDCl_3_): δ 2.54 (s, 3H), 6.08 (s, 1H), 6.87 (d, *J* = 8.4 Hz, 2H), 7.89 (d, *J* = 8.4 Hz, 2H). Data are in good agreement with the literature [45].

*Propiophenone* (**2i**). Following the general procedure, the title compound was obtained (119 mg, 89%). Oil; TLC (Et_2_O/hexanes (1:2)) *R f*= 0.58; ^1^H-NMR (CDCl_3_): δ 1.20 (t, *J* = 7.5 Hz, 3H), 2.97 (q, *J* = 7.5 Hz, 2H), 7.40–7.53 (m, 3H), 7.93 (d, *J* = 7.8 Hz, 2H). Data are in good agreement with the literature [45].

*1,2-Diphenylethanone* (**2j**). Following the general procedure, the title compound was obtained (190 mg, 97%). Oil; TLC (Et_2_O/hexanes (1:2)) *R f*= 0.63; ^1^H-NMR (CDCl_3_): δ 4.28 (s, 2H), 7.25–7.35 (m, 5H), 7.42–7.57 (m, 3 H), 8.00 (t, *J* = 2.1 Hz, 2H). Data are in good agreement with the literature [47].

*1-(Naphthalen-2-yl)ethanone* (**2k**). Following the general procedure, the title compound was obtained (165 mg, 97%). Oil; TLC (Et_2_O/hexanes (1:4)) *R f*= 0.45; ^1^H-NMR (CDCl_3_): δ 2.71 (s, 3H), 7.54–7.59 (m, 2H), 7.85–8.03 (m, 4H), 8.45 (s, 1H). Data are in good agreement with the literature [40].

*5-Methoxy-3,4-dihydronaphthalen-1(2H)-one* (**2l**). Following the general procedure, the title compound was obtained (174 mg, 99%). Oil; TLC (Et_2_O/hexanes (1:2)) *R f*= 0.30; ^1^H-NMR (CDCl_3_): δ 2.05–2.15 (m, 2H), 2.61–2.65 (m, 2H), 2.87 (t, *J* = 6.3 Hz, 2H), 3.84 (s, 3H), 7.01 (d, *J* = 7.8 Hz, 1H), 7.24 (d, *J* = 7.8 Hz, 1H), 7.61 (d, *J* = 7.8 Hz, 1H). Data are in good agreement with the literature [48].

*1-(Benzofuran-2-yl)ethanone* (**2m**). Following the general procedure, the title compound was obtained (133 mg, 83%). Oil; TLC (Et_2_O/hexanes (1:2)) *R f*= 0.40; ^1^H-NMR (CDCl_3_): δ 2.59 (s, 3H), 7.24–7.32 (m, 1H), 7.45–7.49 (m, 2H), 7.54–7.58 (m, 1H), 7.67–7.71 (m, 1H). Data are in good agreement with the literature [49].

*Cyclohexanone* (**2n**). Following the general procedure, the title compound was obtained (89 mg, 91%). Oil; TLC (Et_2_O/hexanes (1:2)) *R f*= 0.45; ^1^H-NMR (CDCl_3_): δ 1.64–1.87 (m, 6H), 2.28–2.32 (m, 4H). Data are in good agreement with the literature [50].

*1-Cyclohexenylethanone* (**2o**). Following the general procedure, the title compound was obtained (114 mg, 92%). Oil; TLC (Et_2_O/hexanes (1:2)) *R f*= 0.53; ^1^H-NMR (CDCl_3_): δ 1.58–1.61 (m, 4H), 2.19–2.24 (m, 4H), 2.25 (s, 3H), 6.85–6.88 (m, 1H). Data are in good agreement with the literature [51].

### 3.3. General Procedure for SnCl_2_/TiCl_3_-Mediated Deoximation of Aldoximes in an Aqueous Solvent

Tin chloride dihydrate (230 mg, 1.0 mmol) and titanium(III) chloride, (0.64 mL, 1.0 mmol, 20% in 3% hydrochloric acid) were added successively to a solution of aldoxime **1** (1.0 mmol) in H_2_O (2 mL) or THF/water (1/3, 2 mL) at rt. The mixture was stirred at ambient temperature until all the starting material was consumed, the solution was extracted with Et_2_O (3 × 5 mL). The combined organic layers were dried over MgSO_4_, filtered, and concentrated *in vacuo*, giving a residue which was subjected to silica gel chromatography to furnish the pure aldehyde **2**.

*Benzaldehyde* (**2p**). Following the general procedure, the title compound was obtained (102 mg, 96%). Oil; TLC (Et_2_O/hexanes (1:2)) *R f*= 0.63; ^1^H-NMR (CDCl_3_): δ 7.47–7.52 (m, 2H), 7.57–7.63 (m, 1H), 7.83–7.87 (m, 2H), 9.99 (s, 1H). Data are in good agreement with the literature [52].

*4-Chlorobenzaldehyde* (**2q**). Following the general procedure, the title compound was obtained (131 mg, 93%). Oil; TLC (Et_2_O/hexanes (1:2)) *R f*= 0.45; ^1^H-NMR (CDCl_3_): δ 7.48 (d, *J* = 9.0 Hz, 2H), 7.80 (d, *J* = 9.0 Hz, 2H), 9.96 (s, 1H). Data are in good agreement with the literature [52].

*3,5-Dimethoxybenzaldehyde* (**2r**). Following the general procedure, the title compound was obtained (161 mg, 97%). Oil; TLC (Et_2_O/hexanes (1:2)) *R f*= 0.43; ^1^H-NMR (CDCl_3_): δ 3.64 (s, 3H), 3.65 (s, 3H), 6.51 (d, *J* = 2.4 Hz, 1H), 6.81 (d, *J* = 2.4 Hz, 2H), 9.70 (s, 1H). Data are in good agreement with the literature [53].

## 4. Conclusions

In summary, we report a simple, mild and inexpensive method for deoximation of oximes in an aqueous solvent. This procedure appears to be advantageous in terms of chemical yield, reaction conditions, and functional group compatibility.

## Supplementary Materials

Supplementary materials can be accessed at: http://www.mdpi.com/1420-3049/17/3/2464/s1.
