# Supplementary material for: SnCl2/TiCl3-Mediated Deoximation of Oximes in an Aqueous Solvent
Source: Molecules. 2012 Mar 1;17(3):2464–73. doi: 10.3390/molecules17032464 (PMC6268544; doi:10.3390/molecules17032464)
Supplement: Supplementary file 1 [file molecules-17-02464-s001.pdf]

Supplementary Materials

$SnCl_2/TiCl_3$-Mediated Deoximation of Ketoximes in an Aqueous Solvent

Mei-Huey Lin *, Han-Jun Liu, Cheng-YuChang, Wei-Cheng Lin and Tsung-Hsun Chuang

Department of Chemistry, National Changhua University of Education, Changhua, Taiwan

Table of Contents:

Copies of ^{1}H NMR spectra. **Page** S2-S19

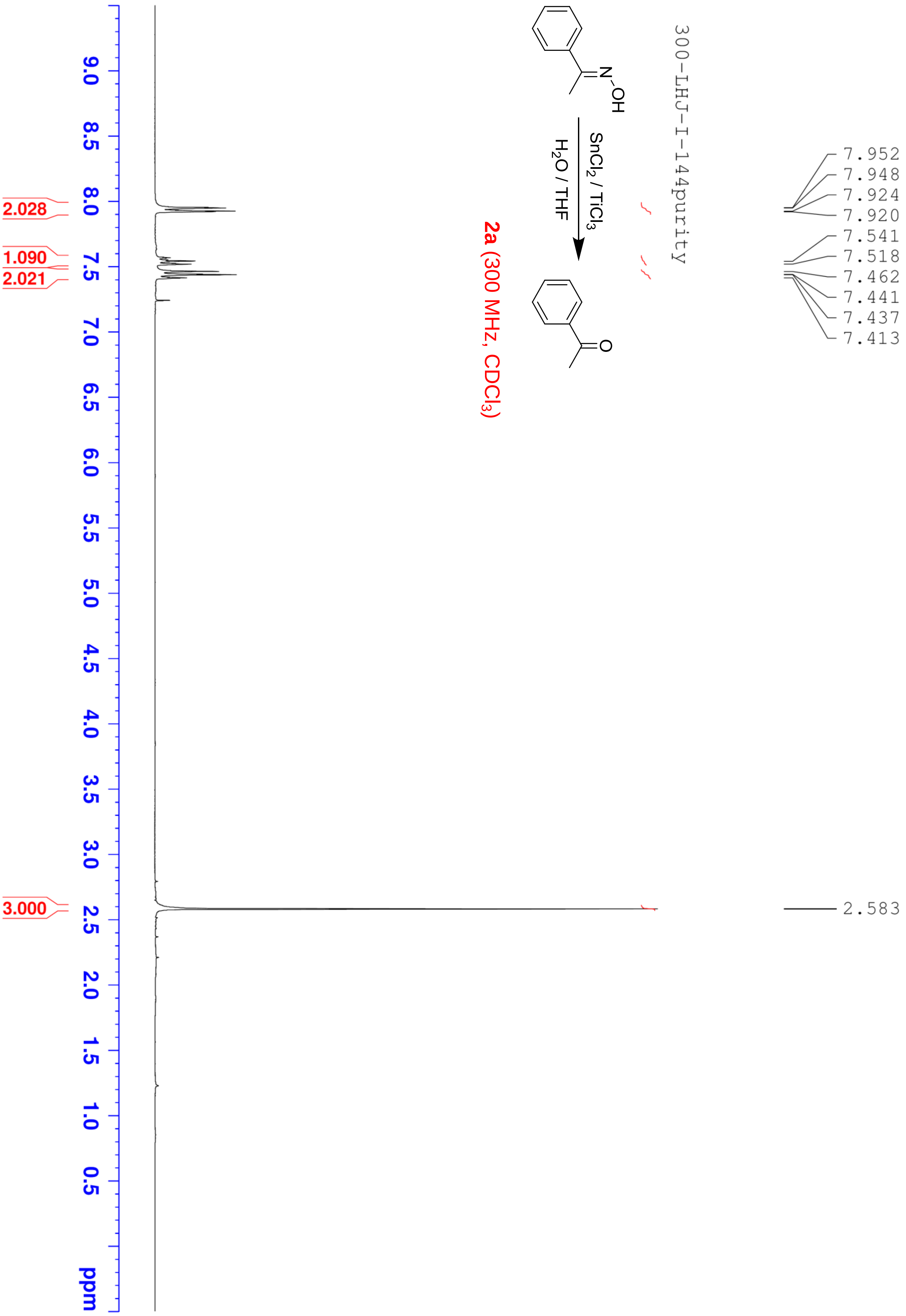

7.952
7.948
7.924
7.920
7.541
7.518
7.462
7.441
7.437
7.413
2.583
300-LHJ-I-144purity
N
OH
SnCl2 / TiCl3
H2O / THF
O
2a (300 MHz, CDCl3)
9.0
8.5
8.0
7.5
7.0
6.5
6.0
5.5
5.0
4.5
4.0
3.5
3.0
2.5
2.0
1.5
1.0
0.5
ppm
2.028
1.090
2.021
3.000

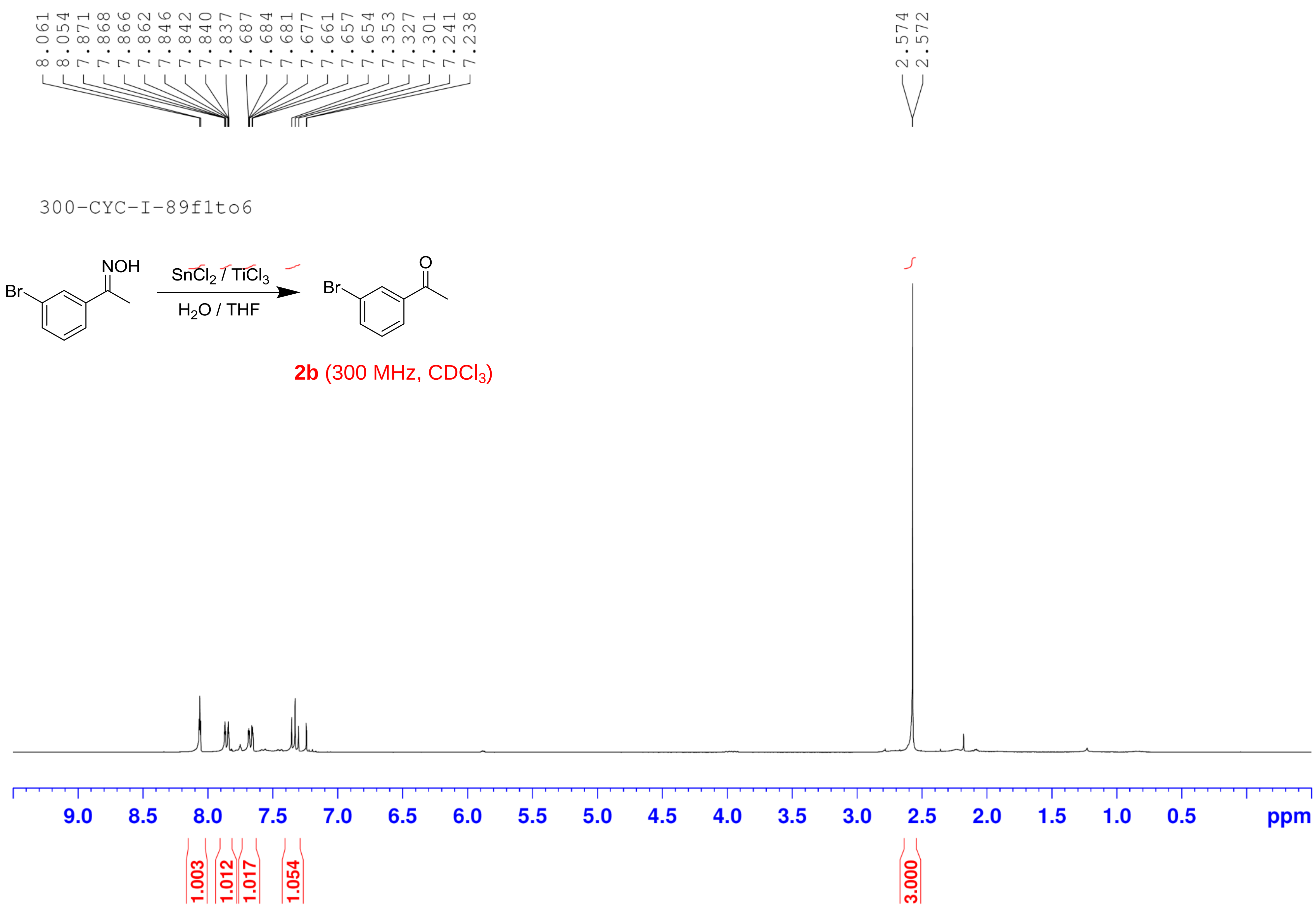
8.061
8.054
7.871
7.868
7.866
7.862
7.846
7.842
7.840
7.837
7.687
7.684
7.681
7.677
7.661
7.657
7.654
7.353
7.327
7.301
7.241
7.238
2.574
2.572
300-CYC-I-89f1to6
NOH
Br
SnCl2 / TiCl3
H2O / THF
O
2b (300 MHz, CDCl3)
9.0
8.5
8.0
7.5
7.0
6.5
6.0
5.5
5.0
4.5
4.0
3.5
3.0
2.5
2.0
1.5
1.0
0.5
ppm
1.003
1.012
1.017
1.054
3.000

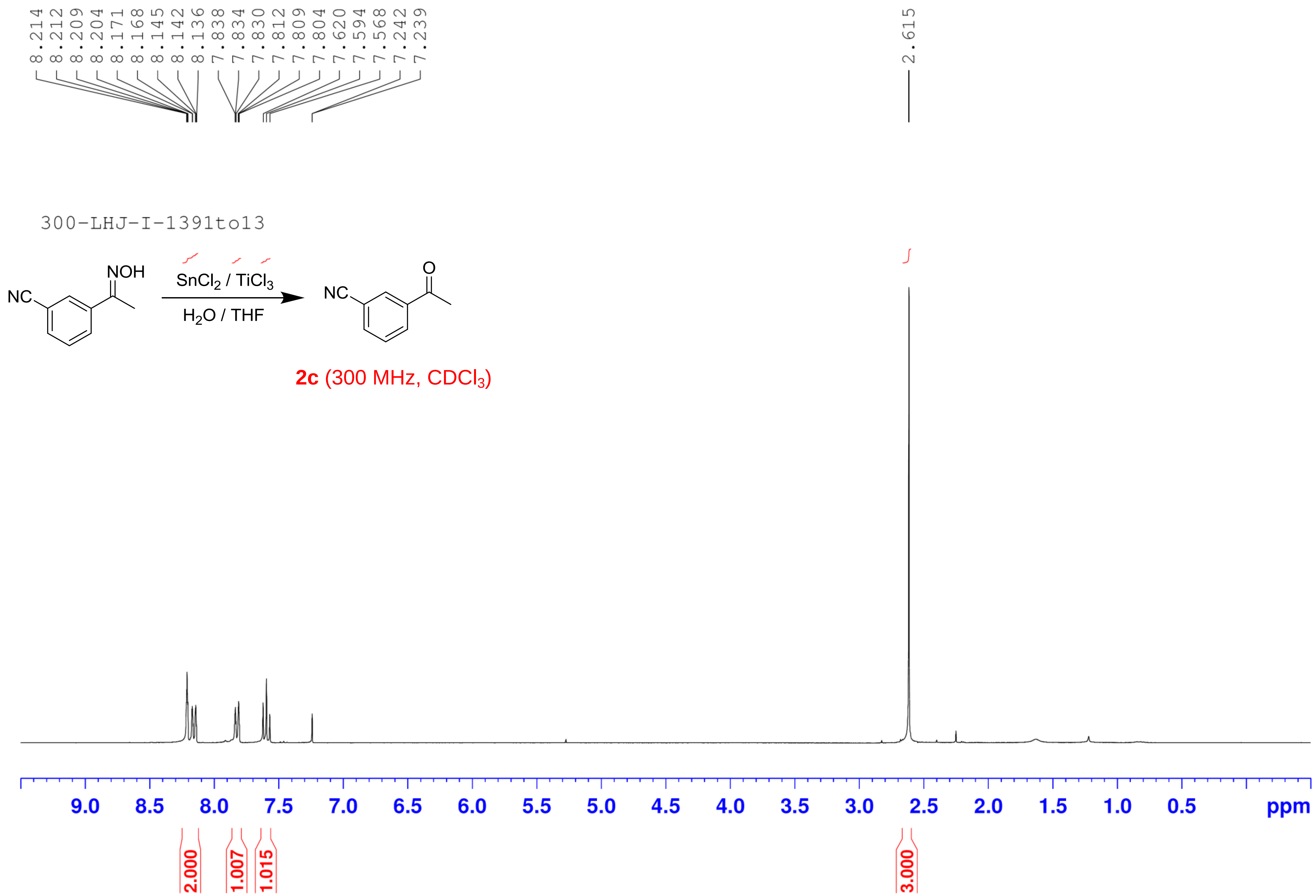
8.214
8.212
8.209
8.204
8.171
8.168
8.145
8.142
8.136
7.838
7.834
7.830
7.812
7.809
7.804
7.620
7.594
7.568
7.242
7.239
2.615
300-LHJ-I-1391to13
NOH
NC
$SnCl_2$ / $TiCl_3$
H_2O / THF
O
NC
2c (300 MHz, $CDCl_3$)
9.0
8.5
8.0
7.5
7.0
6.5
6.0
5.5
5.0
4.5
4.0
3.5
3.0
2.5
2.0
1.5
1.0
0.5
ppm
2.000
1.007
1.015
3.000

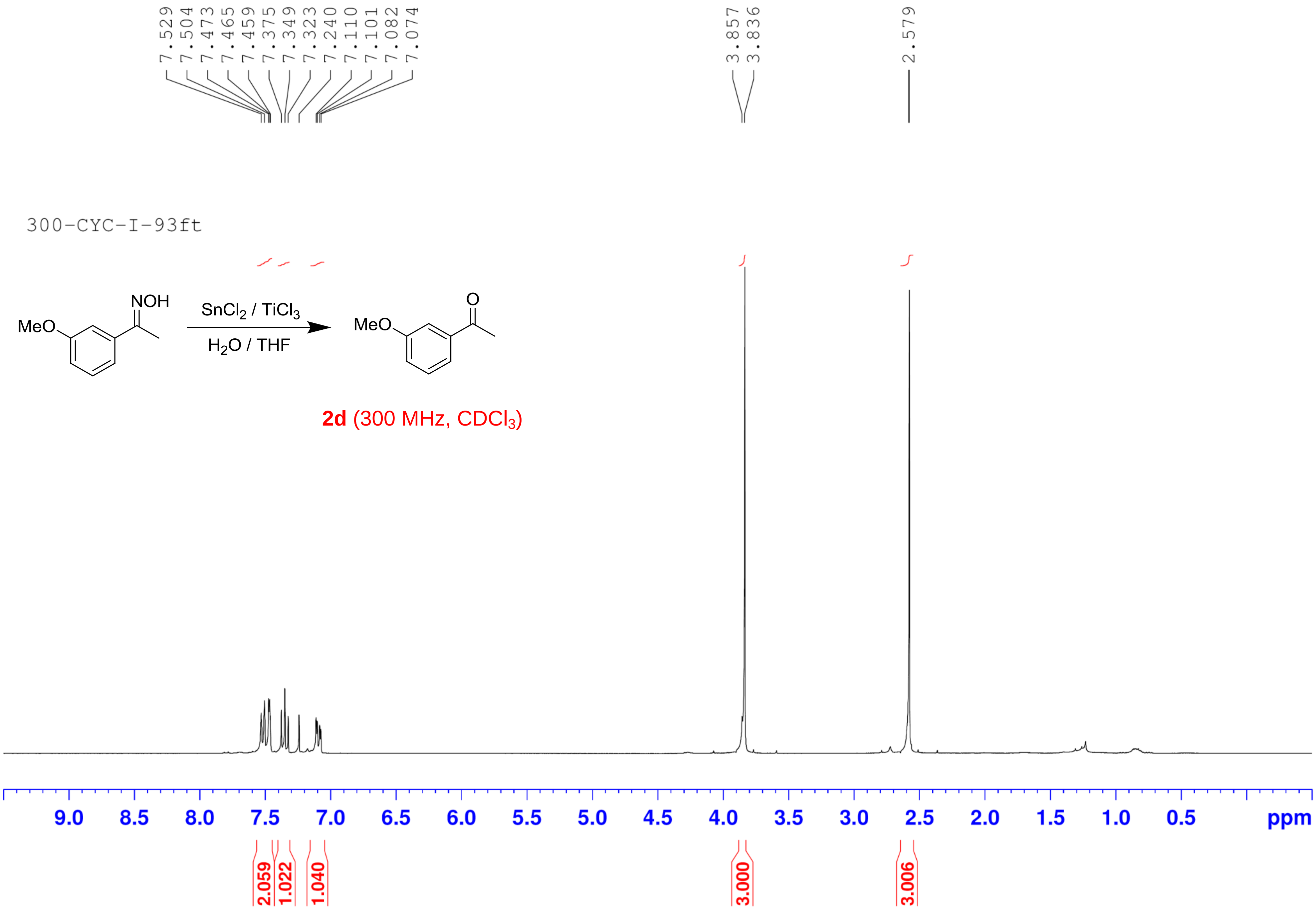
7.529
7.504
7.473
7.465
7.459
7.375
7.349
7.323
7.240
7.110
7.101
7.082
7.074
3.857
3.836
2.579
300-CYC-I-93ft
MeO
NOH
SnCl2 / TiCl3
H2O / THF
O
2d (300 MHz, CDCl3)
9.0
8.5
8.0
7.5
7.0
6.5
6.0
5.5
5.0
4.5
4.0
3.5
3.0
2.5
2.0
1.5
1.0
0.5
ppm
2.059
1.022
1.040
3.000
3.006

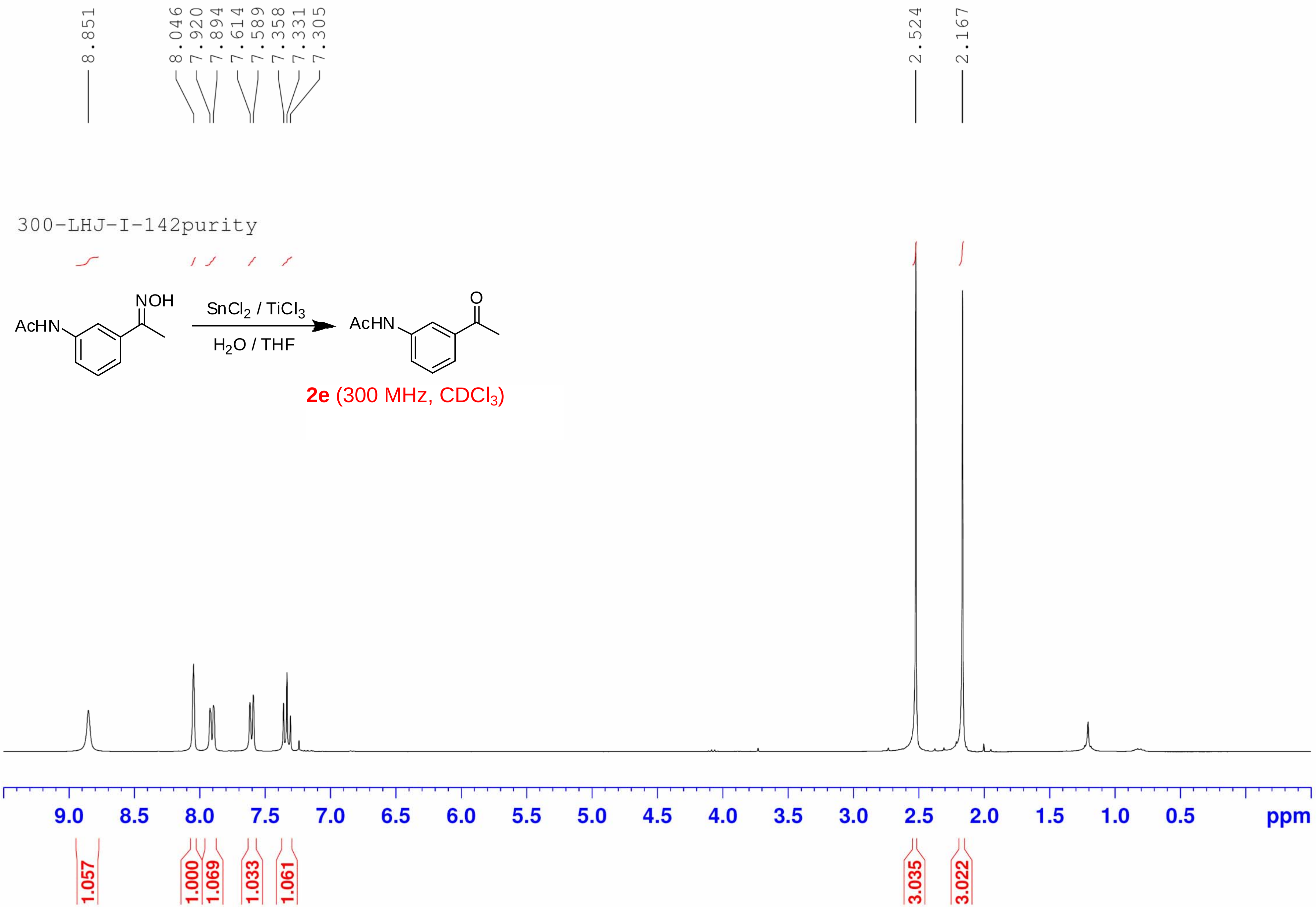

2e (300 MHz, $CDCl_3$)

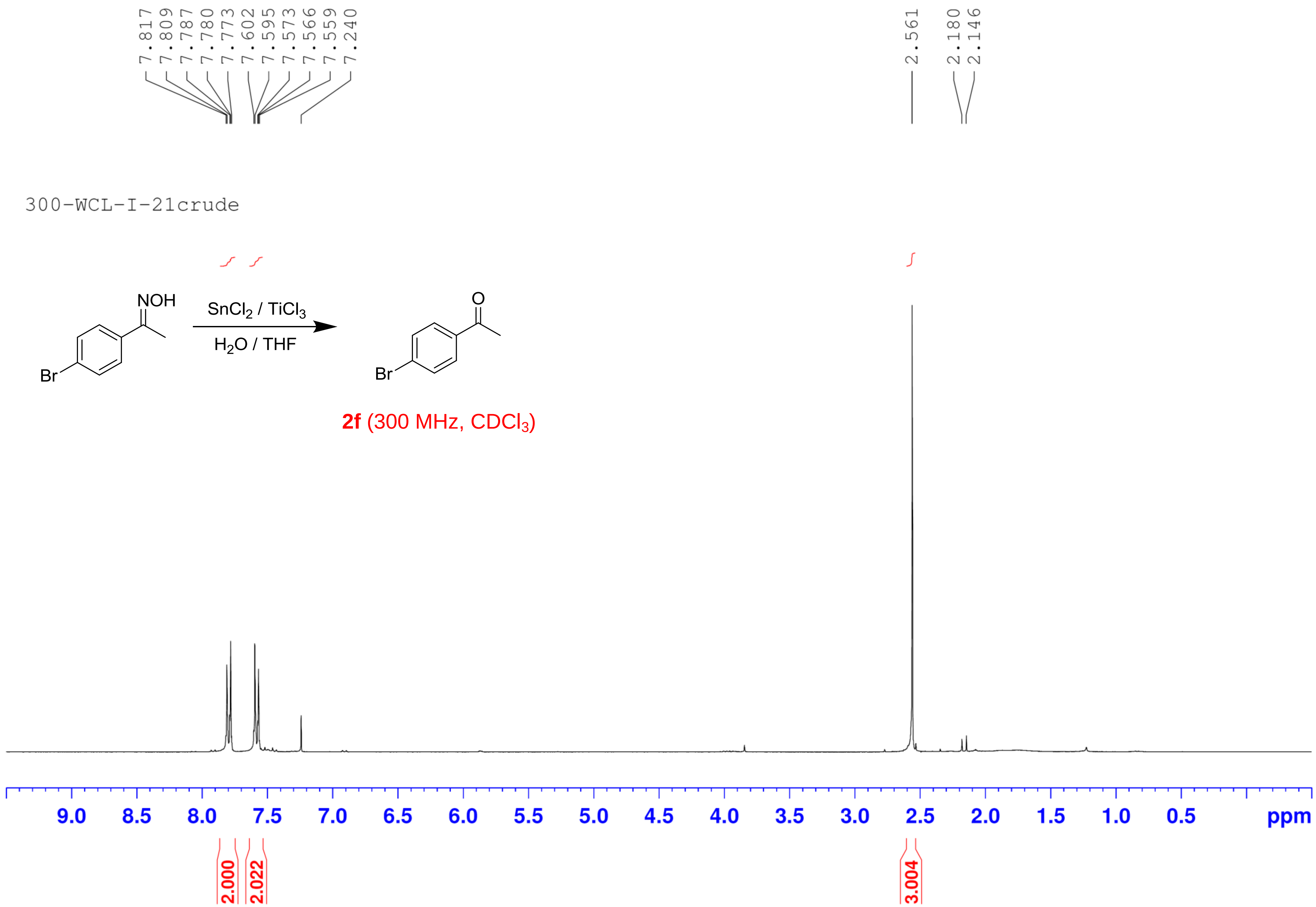

2f (300 MHz, $CDCl_3$)

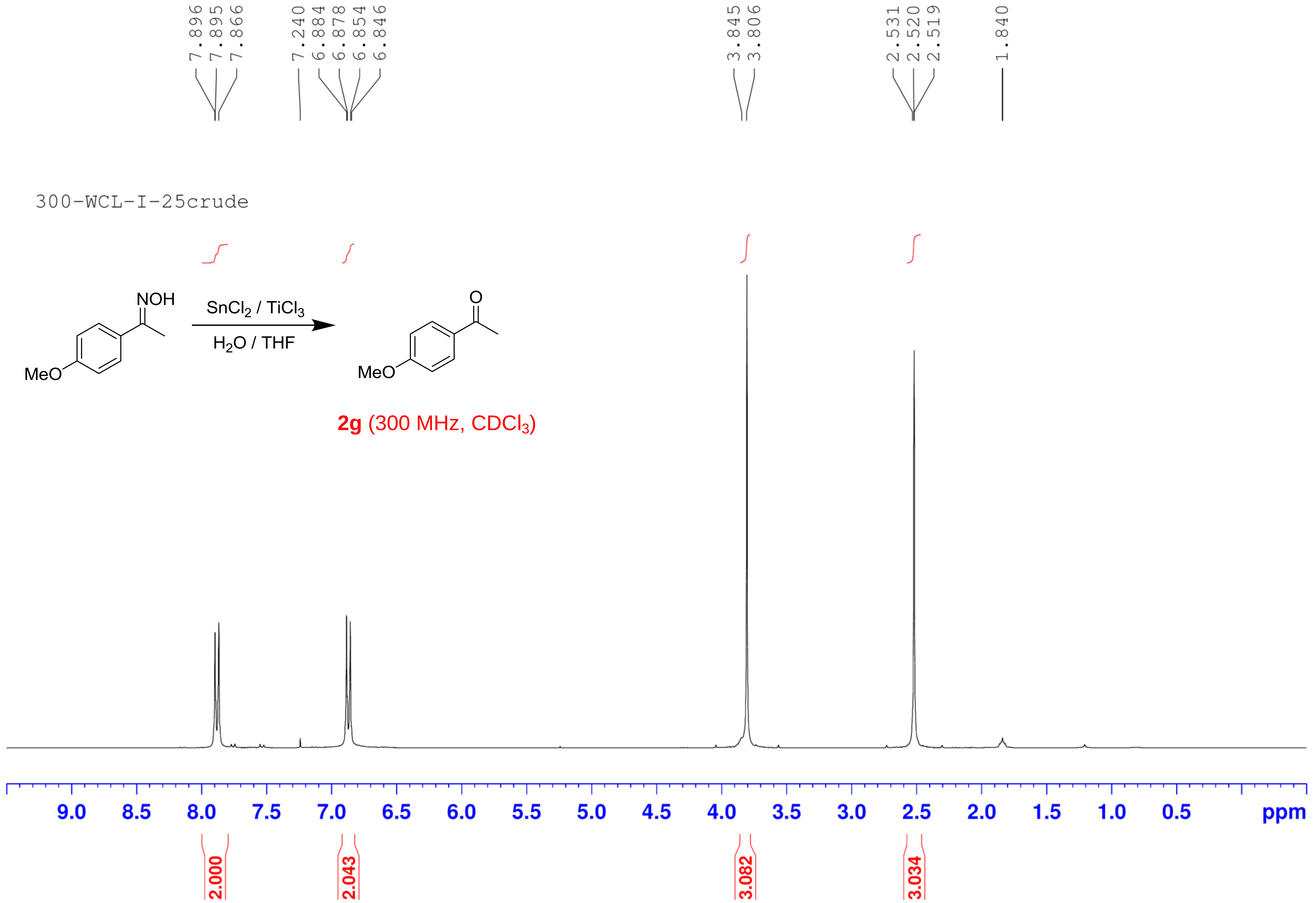

2g (300 MHz, $CDCl_3$)

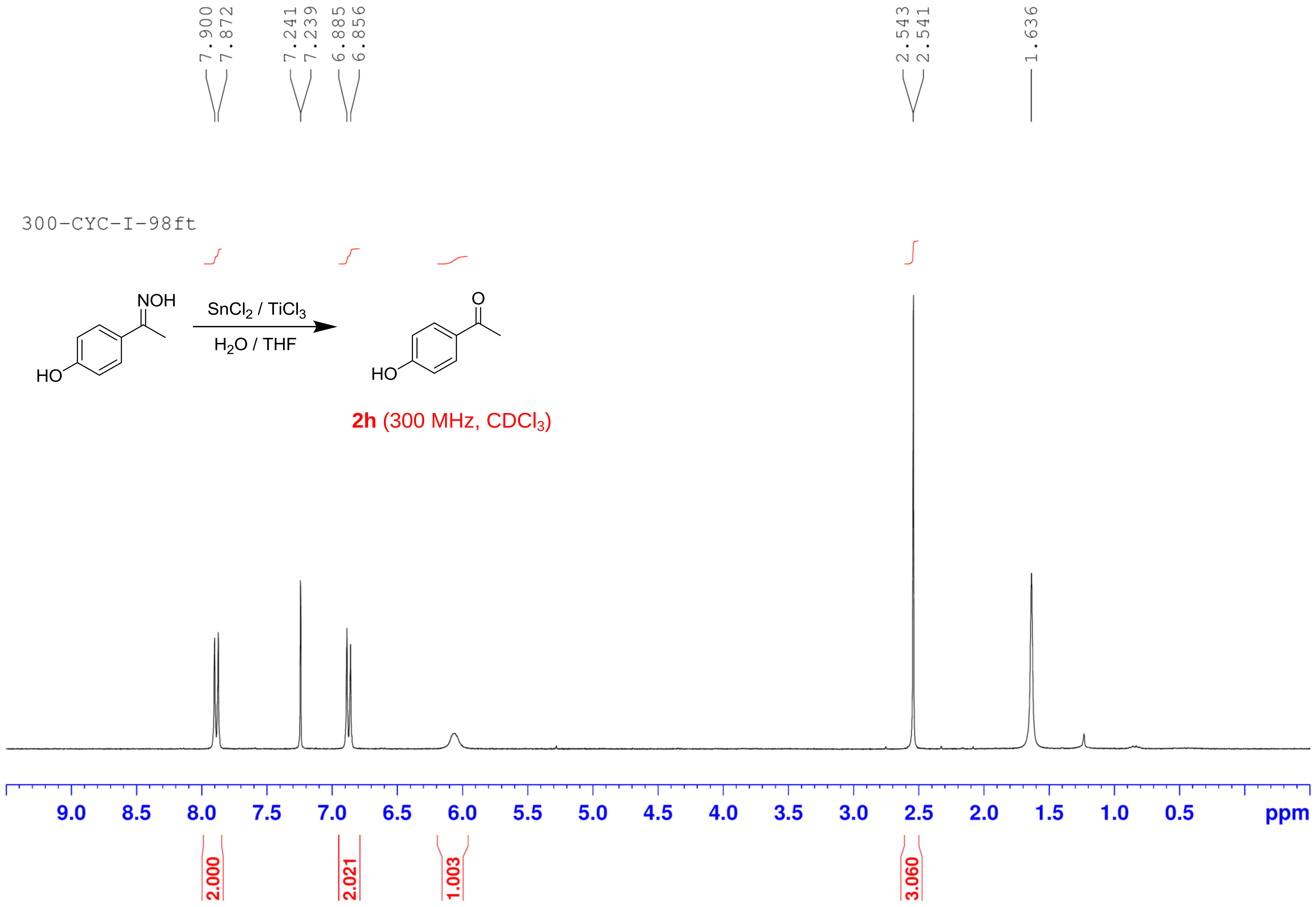
7.900
7.872
7.241
7.239
6.885
6.856
2.543
2.541
1.636
300-CYC-I-98ft
NOH
HO
$SnCl_2$ / $TiCl_3$
H_2O / THF
O
HO
2h (300 MHz, $CDCl_3$)
9.0
8.5
8.0
7.5
7.0
6.5
6.0
5.5
5.0
4.5
4.0
3.5
3.0
2.5
2.0
1.5
1.0
0.5
ppm
2.000
2.021
1.003
3.060

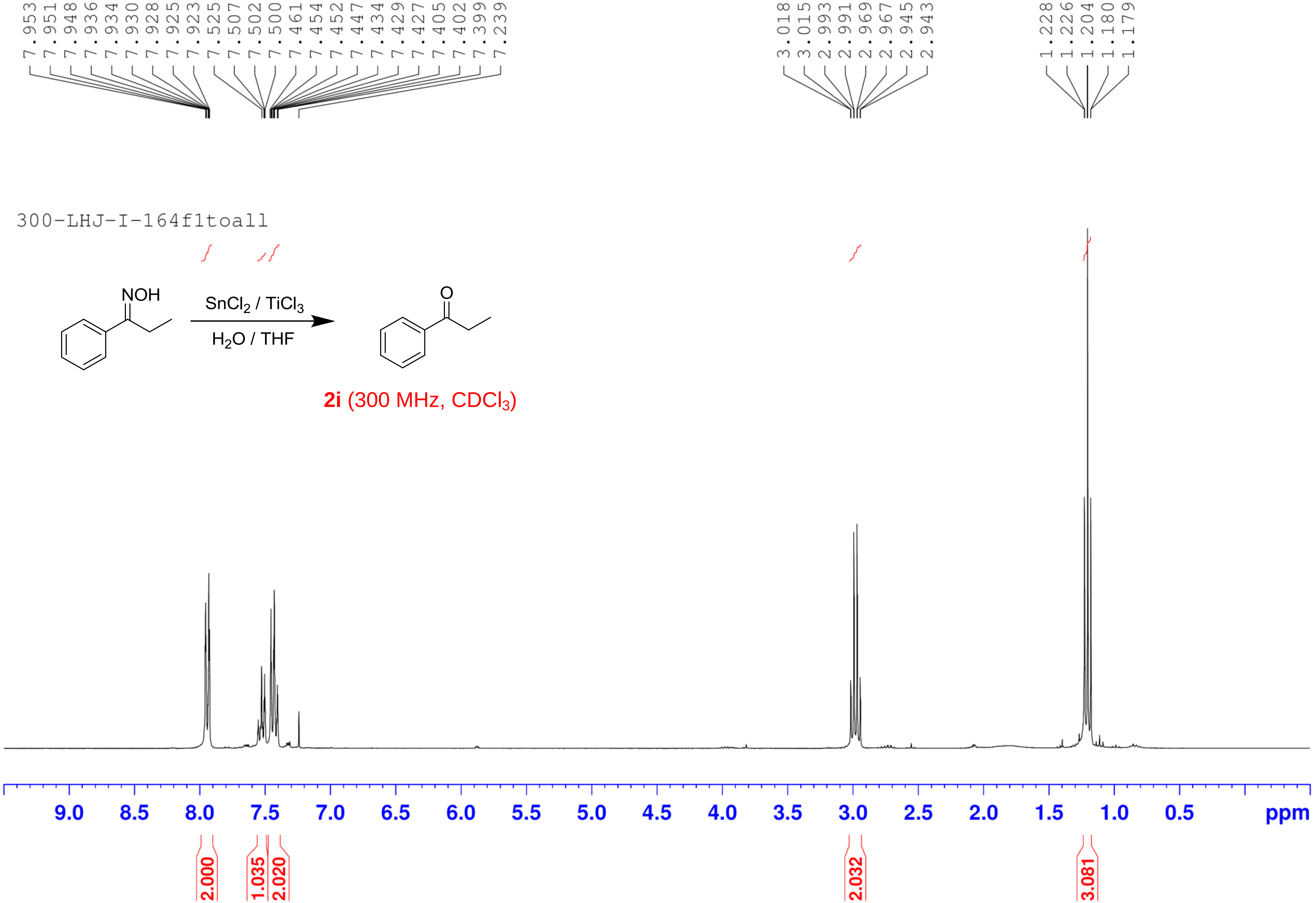

7.953
7.951
7.948
7.936
7.934
7.930
7.928
7.925
7.923
7.525
7.507
7.502
7.500
7.461
7.454
7.452
7.447
7.434
7.429
7.427
7.405
7.402
7.399
7.239
3.018
3.015
2.993
2.991
2.969
2.967
2.945
2.943
1.228
1.226
1.204
1.180
1.179
300-LHJ-I-164f1toall
NOH
SnCl2 / TiCl3
H2O / THF
O
2i (300 MHz, CDCl3)
9.0
8.5
8.0
7.5
7.0
6.5
6.0
5.5
5.0
4.5
4.0
3.5
3.0
2.5
2.0
1.5
1.0
0.5
ppm
2.000
1.035
2.020
2.032
3.081

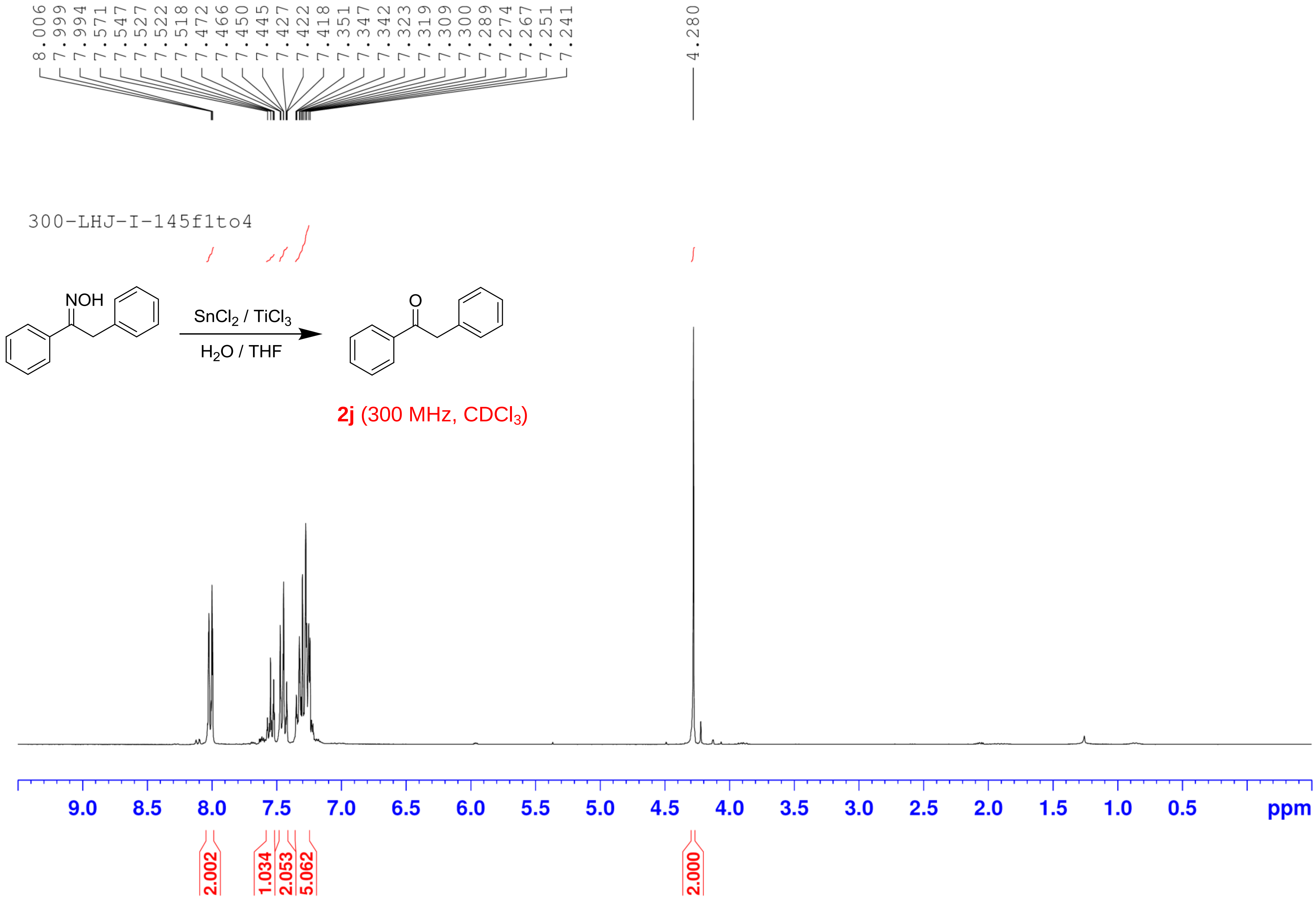
8.006
7.999
7.994
7.571
7.547
7.527
7.522
7.518
7.472
7.466
7.450
7.445
7.427
7.422
7.418
7.351
7.347
7.342
7.323
7.319
7.309
7.300
7.289
7.274
7.267
7.251
7.241
4.280
300-LHJ-I-145f1to4
NOH
$SnCl_2$ / $TiCl_3$
H_2O / THF
O
2j (300 MHz, $CDCl_3$)
9.0
8.5
8.0
7.5
7.0
6.5
6.0
5.5
5.0
4.5
4.0
3.5
3.0
2.5
2.0
1.5
1.0
0.5
ppm
2.002
1.034
2.053
5.062
2.000

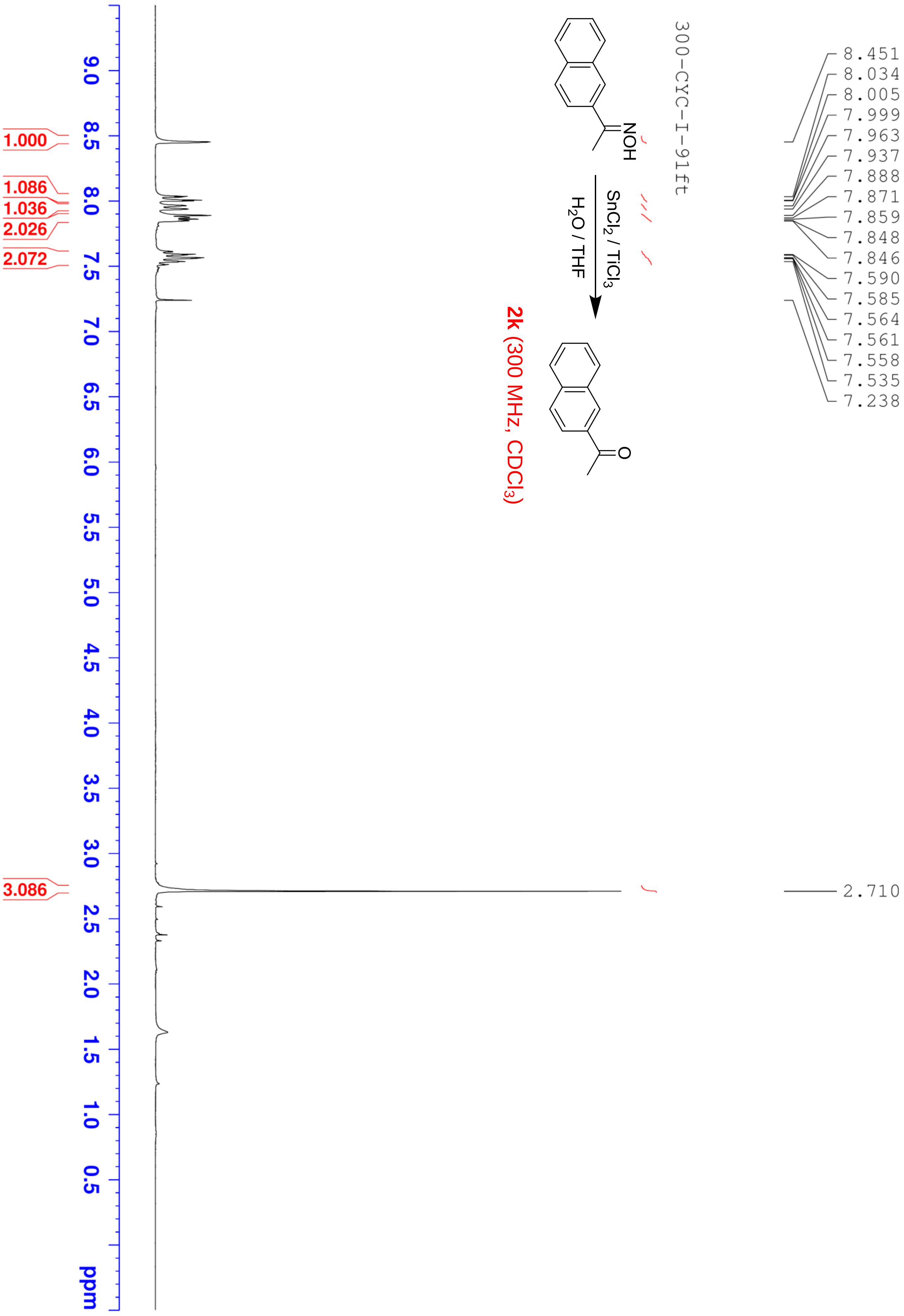

300-CYC-I-91ft
NOH
SnCl2 / TiCl3
H2O / THF
O
2k (300 MHz, CDCl3)
8.451
8.034
8.005
7.999
7.963
7.937
7.888
7.871
7.859
7.848
7.846
7.590
7.585
7.564
7.561
7.558
7.535
7.238
2.710
1.000
1.086
1.036
2.026
2.072
3.086
9.0
8.5
8.0
7.5
7.0
6.5
6.0
5.5
5.0
4.5
4.0
3.5
3.0
2.5
2.0
1.5
1.0
0.5
ppm

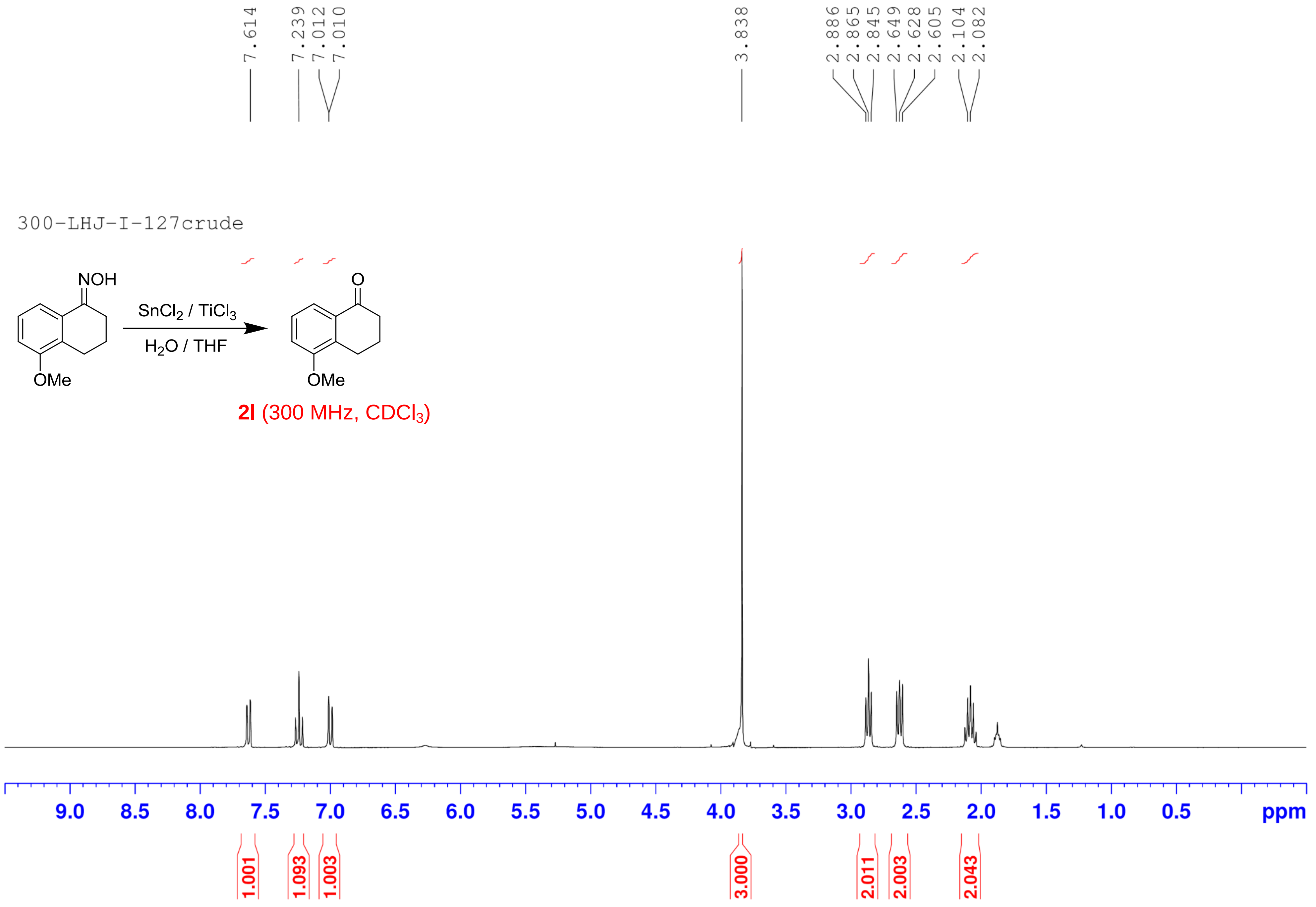
7.614
7.239
7.012
7.010
3.838
2.886
2.865
2.845
2.649
2.628
2.605
2.104
2.082
300-LHJ-I-127crude
NOH
SnCl2 / TiCl3
H2O / THF
OMe
O
OMe
2l (300 MHz, CDCl3)
9.0
8.5
8.0
7.5
7.0
6.5
6.0
5.5
5.0
4.5
4.0
3.5
3.0
2.5
2.0
1.5
1.0
0.5
ppm
1.001
1.093
1.003
3.000
2.011
2.003
2.043

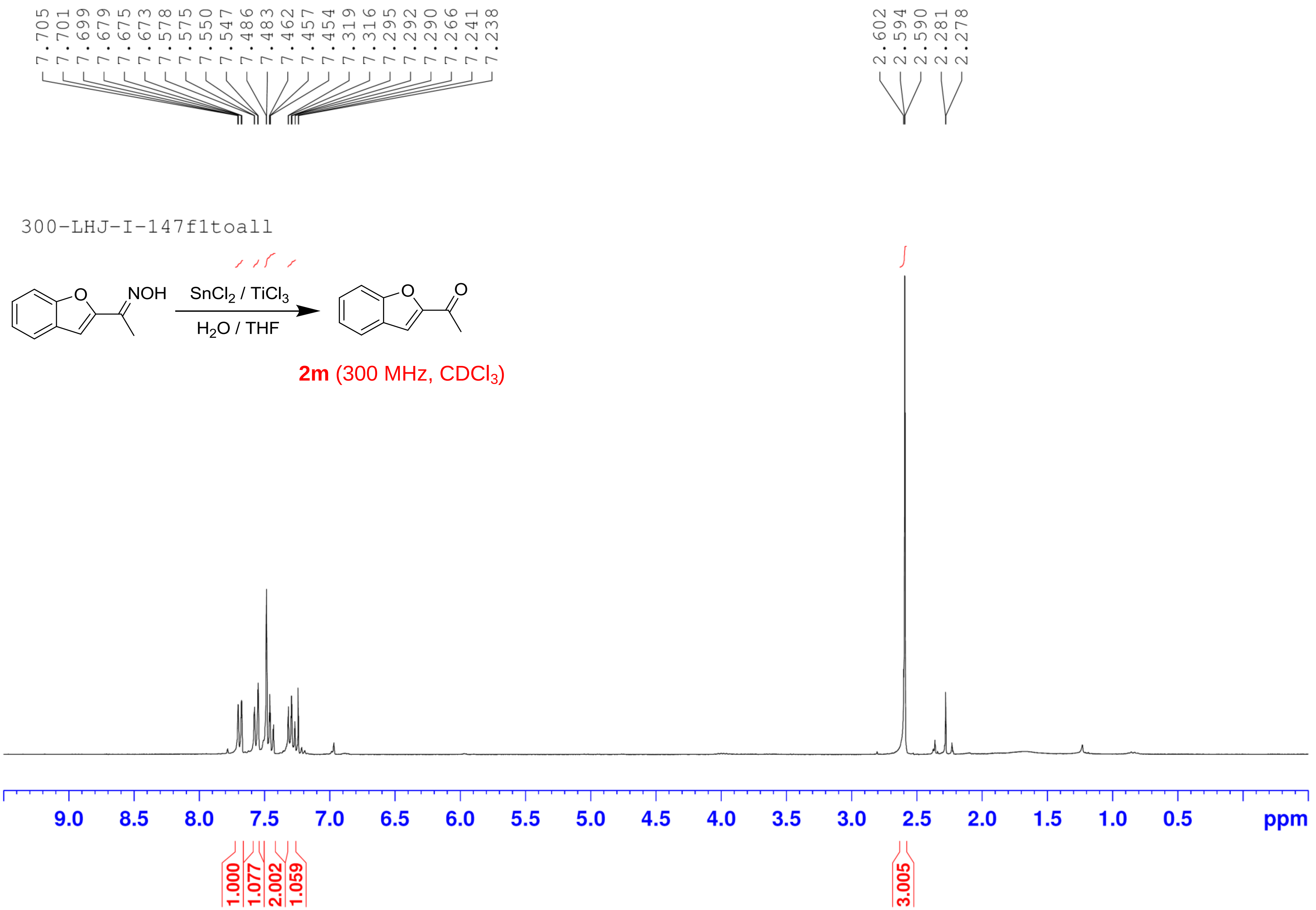
7.705
7.701
7.699
7.679
7.675
7.673
7.578
7.575
7.550
7.547
7.486
7.483
7.462
7.457
7.454
7.319
7.316
7.295
7.292
7.290
7.266
7.241
7.238
2.602
2.594
2.590
2.281
2.278
300-LHJ-I-147f1toall
NOH
O
$SnCl_2$ / $TiCl_3$
H_2O / THF
O
O
2m (300 MHz, $CDCl_3$)
9.0
8.5
8.0
7.5
7.0
6.5
6.0
5.5
5.0
4.5
4.0
3.5
3.0
2.5
2.0
1.5
1.0
0.5
ppm
1.000
1.077
2.002
1.059
3.005

300-LHJ-II-8purity

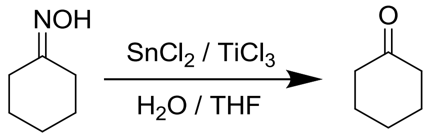

2n (300 MHz, $CDCl_3$)

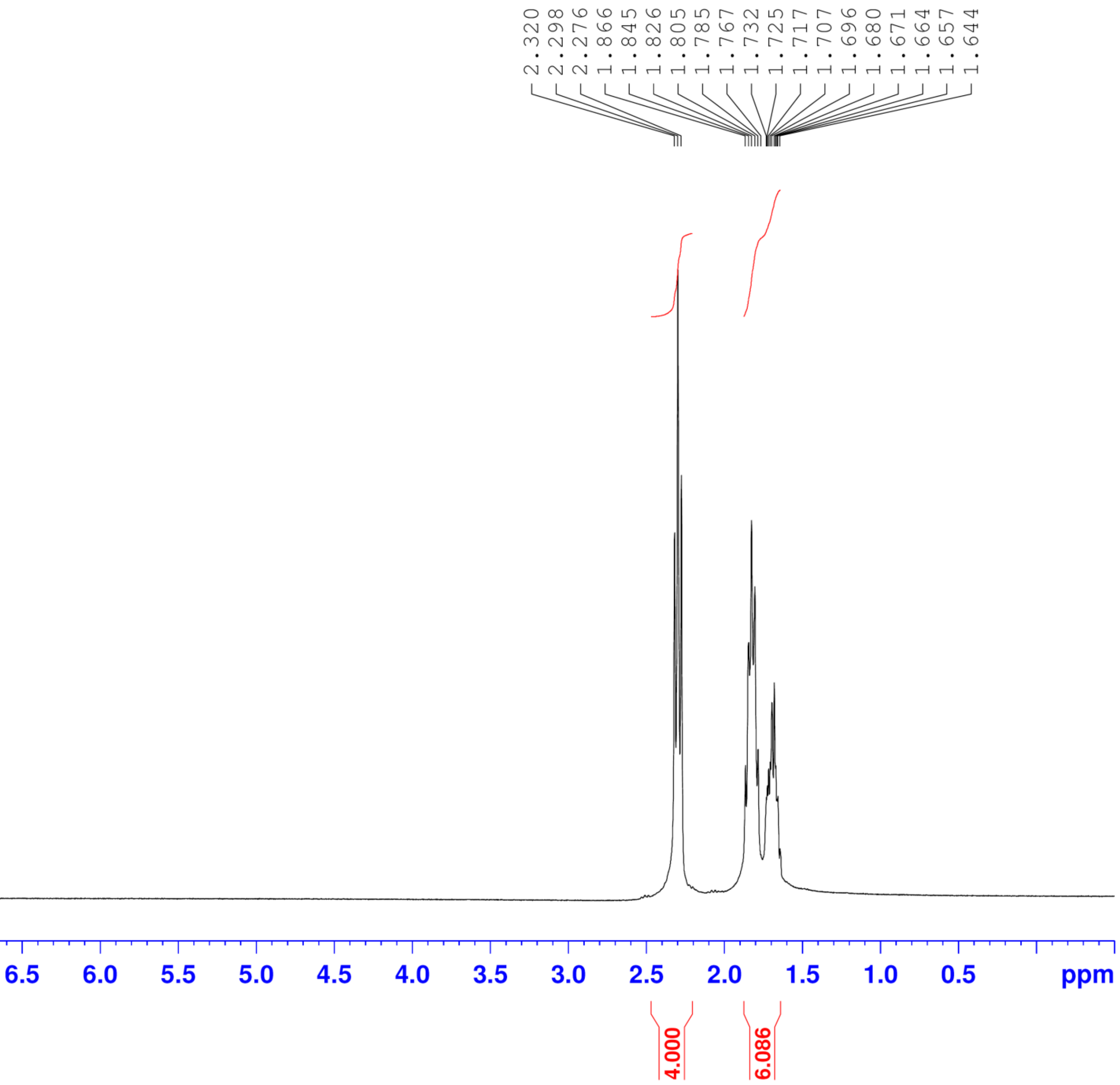

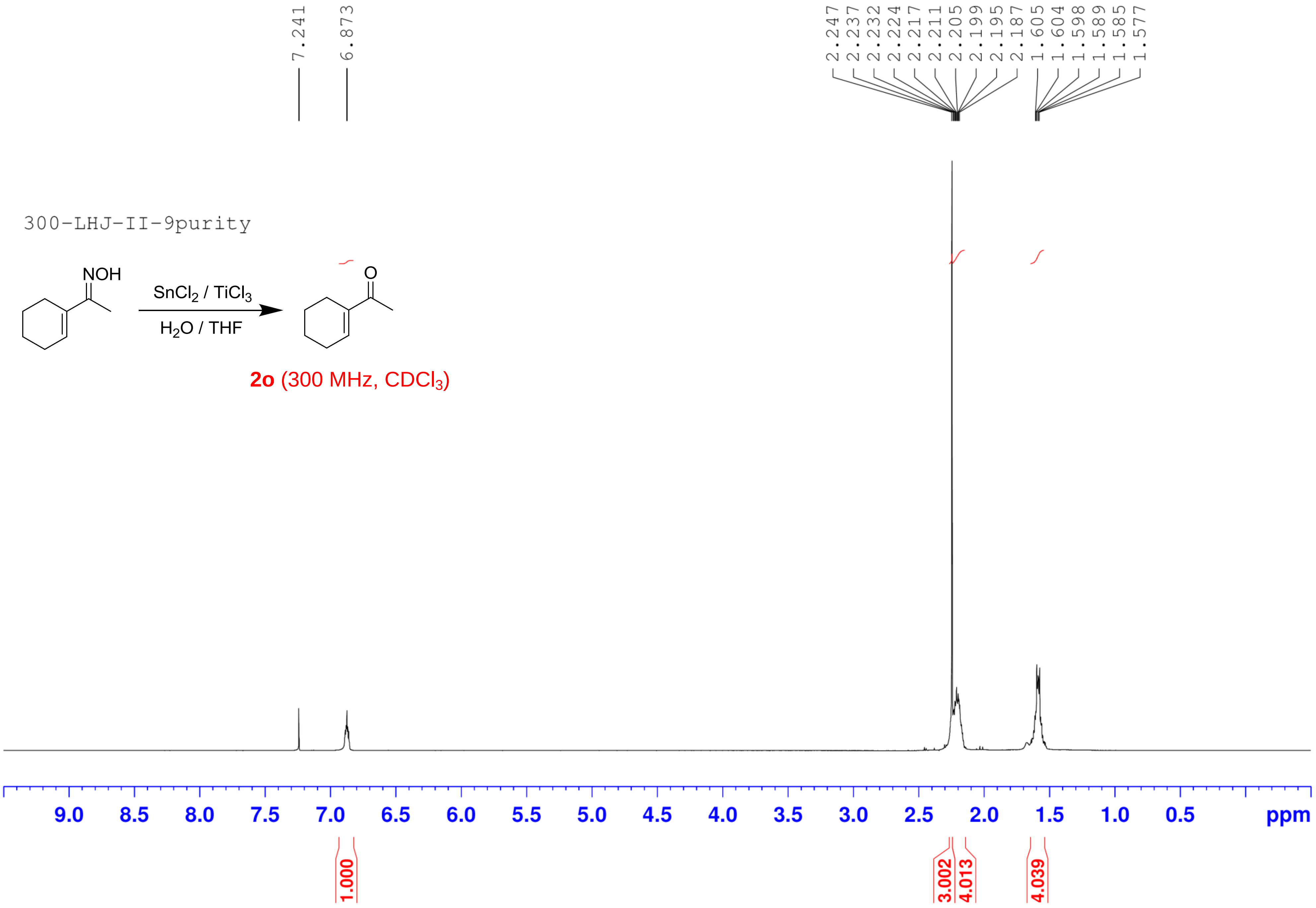
7.241
6.873
2.247
2.237
2.232
2.224
2.217
2.211
2.205
2.199
2.195
2.187
1.605
1.604
1.598
1.589
1.585
1.577
300-LHJ-II-9purity
NOH
$SnCl_2$ / $TiCl_3$
H_2O / THF
O
2o (300 MHz, $CDCl_3$)
9.0
8.5
8.0
7.5
7.0
6.5
6.0
5.5
5.0
4.5
4.0
3.5
3.0
2.5
2.0
1.5
1.0
0.5
ppm
1.000
3.002
4.013
4.039

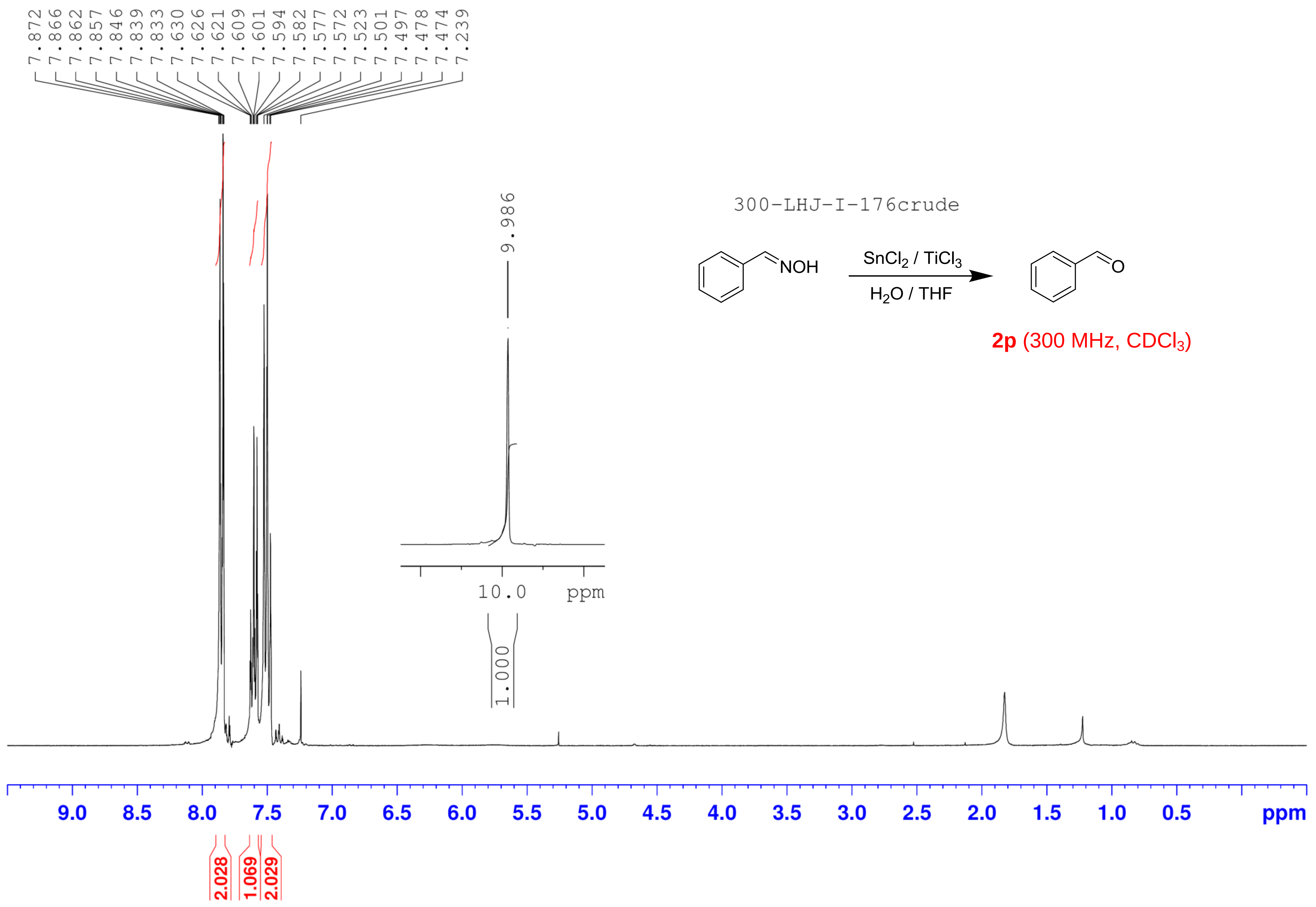
7.872
7.866
7.862
7.857
7.846
7.839
7.833
7.630
7.626
7.621
7.609
7.601
7.594
7.582
7.577
7.572
7.523
7.501
7.497
7.478
7.474
7.239
9.986
10.0
ppm
1.000
300-LHJ-I-176crude
NOH
$SnCl_2$ / $TiCl_3$
H_2O / THF
O
2p (300 MHz, $CDCl_3$)
9.0
8.5
8.0
7.5
7.0
6.5
6.0
5.5
5.0
4.5
4.0
3.5
3.0
2.5
2.0
1.5
1.0
0.5
ppm
2.028
1.069
2.029

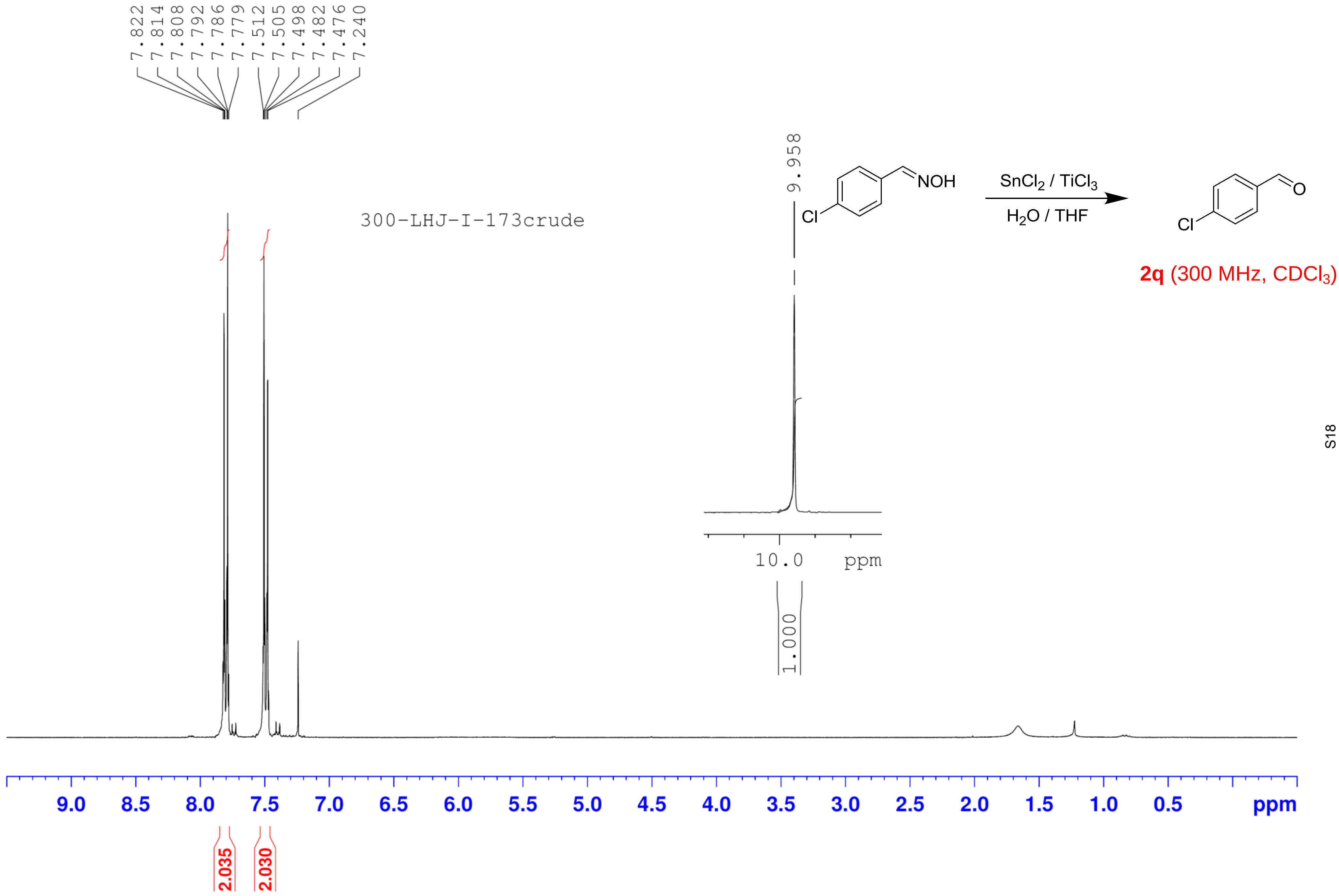

2q (300 MHz, $CDCl_3$)

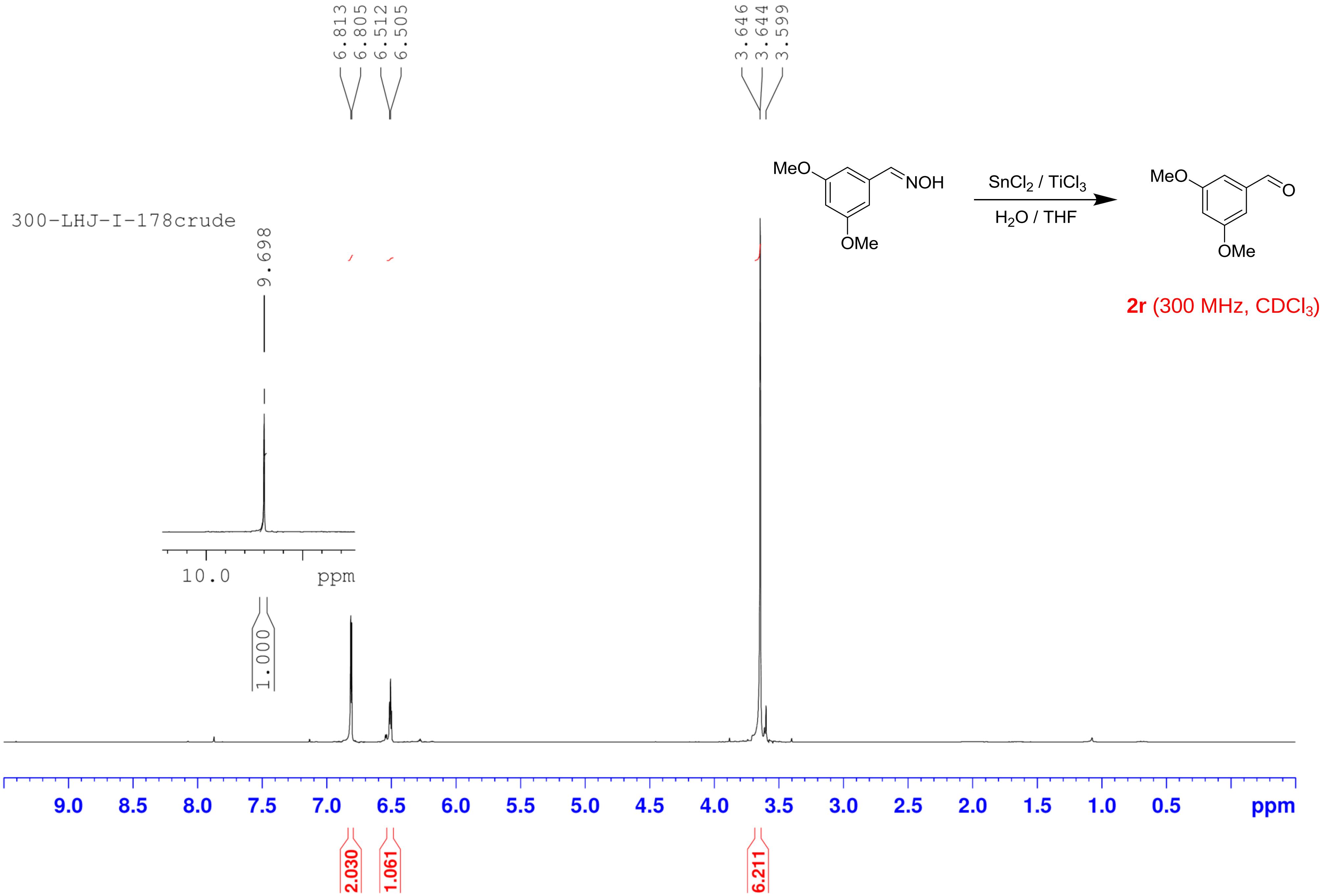

2r (300 MHz, $CDCl_3$)
